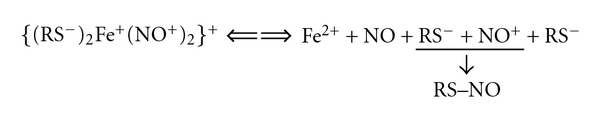# Electronic and Spatial Structures of Water-Soluble Dinitrosyl Iron Complexes with Thiol-Containing Ligands Underlying Their Ability to Act as Nitric Oxide and Nitrosonium Ion Donors

**DOI:** 10.1155/2011/878236

**Published:** 2012-02-14

**Authors:** Anatoly F. Vanin, Dosymzhan Sh. Burbaev

**Affiliations:** N. N. Semyonov Institute of Chemical Physics, Russian Academy of Sciences, Kosygin Street 4, Moscow 119991, Russia

## Abstract

The ability of mononuclear dinitrosyl iron commplexes (M-DNICs) with thiolate ligands to act as NO donors and to trigger S-nitrosation of thiols can be explain only in the paradigm of the model of the [Fe^+^(NO^+^)_2_] core ({Fe(NO)_2_}^7^ according to the Enemark-Feltham classification). Similarly, the {(RS^−^)_2_Fe^+^(NO^+^)_2_}^+^ structure describing the distribution of unpaired electron density in M-DNIC corresponds to the low-spin (*S* = 1/2) state with a d^7^ electron configuration of the iron atom and predominant localization of the unpaired electron on MO(d_z2_) and the square planar structure of M-DNIC. On the other side, the formation of molecular orbitals of M-DNIC including orbitals of the iron atom, thiolate and nitrosyl ligands results in a transfer of electron density from sulfur atoms to the iron atom and nitrosyl ligands. Under these conditions, the positive charge on the nitrosyl ligands diminishes appreciably, the interaction of the ligands with hydroxyl ions or with thiols slows down and the hydrolysis of nitrosyl ligands and the S-nitrosating effect of the latter are not manifested. Most probably, the S-nitrosating effect of nitrosyl ligands is a result of weak binding of thiolate ligands to the iron atom under conditions favoring destabilization of M-DNIC.

By present, endogenous dinitrosyl iron complexes (DNICs) with thiol-containing ligands, that is, DNICs having a Fe(NO)_2_ core, were detected in a vast majority of animal tissues and cell cultures [1–8]. These DNIC are generated in the presence of nitric oxide (NO) released from endogenous or exogenous sources (L-arginine or nitrite, resp.). In biological systems, DNIC are predominantly represented by protein-bound mononuclear forms with characteristic anisotropic EPR signals at *g*_⊥_ = 2.04 and *g*_||_ = 2.014, commonly termed as “the 2.03 signal” in accordance with the average value of the *g*-factor [9]. The 2.03 signal was recorded at both low and ambient temperatures. One of the first records of the 2.03 signal in yeast cells and animal tissues obtained by three independent groups of investigators in the USSR, Great Britain, and the USA as early as mid-1960s are shown in Figure 1 [10–14].

 Paramagnetic mono- and diamagnetic binuclear forms of low-molecular DNIC (M- and B-DNIC, resp.) can be easily prepared by chemical methods [15]. The simplest procedure for obtaining hydrophilic M-DNIC with thiol-containing ligands, for example, cysteine or glutathione, was developed as early as the 1960–1970s [16, 17] and includes treatment of aqueous solutions of thiols with gaseous NO in the presence of Fe^2+^ ions at neutral pH. Paramagnetic M-DNICs (*g* = 2.03) are formed predominantly at thiol: Fe^2+^ molar ratios above 10, while diamagnetic B-DNICs are generated at thiol: Fe^2+^ ratios below 4 [15].

 Studies into biological activities of these DNIC established their potent vasodilating [18] and hypotensive [19] effects. They effectively suppress platelet aggregation [20], increase red blood cell elasticity [21], and reduce the necrotic zone in animals with experimental myocardial infarction *in vivo* and in isolated animal hearts [22]. DNICs with glutathione increase survival, improve myocardial function, and stimulate microcirculation in animals at hemorrhagic shock [23]. Moreover, DNIC with cysteine or glutathione accelerate skin wound healing [24], improve the function of cavernous tissue, and suppress its fibrous transformation in rats with denervated penis [25, 26]. Moreover, they induce persistent erection of the penis in rats both at innervation and after surgical denervation [25, 26].

 A stable drug form (pharmacological name Oxacom), able to retain its physicochemical and physiological characteristics upon long-term (no less than one year) storage in dry air, was recently synthesized on the basis of DNIC with glutathione and successfully underwent pharmacological testing. Clinical trials on healthy volunteers demonstrated that single (bolus) i/v injection of Oxacom (5 mg of the preparation, namely, 0.2 *μ*moles of DNIC with glutathione/kg of human weight) induced long-lasting hypotension (20% drop of systolic and diastolic pressure), which lasted 10–20 h [27].

 Obviously, peculiar biological activities of low-molecular DNIC with thiol-containing ligands having a Fe(NO)_2_ core can be attributed to their ability to donate neutral NO molecules, which control an immense variety of physiological processes (including the above-mentioned ones) via a cGMP-dependent pathway. Moreover, low-molecular DNIC with thiol-containing ligands initiate S-nitrosation of low-molecular and protein-bound thiols responsible for an immense diversity of biological manifestations of NO activity or, more particularly, its ionized form, namely, nitrosonium ions (NO^+^) (see below).

 Let us consider some facts relevant to the ability of water-soluble DNIC with thiol-containing ligands to act as NO and NO^+^ donors.

 The first evidence supporting the ability of M- and B-DNIC to release NO was obtained in pioneering studies of vasodilating effects of DNIC with cysteine on isolated blood vessels dating back to 1990s [28–31]. This vasodilating effect was accompanied by activation of guanylate cyclase in blood vasculature, most probably, as a result of NO release from DNIC. The inhibiting effect of the NO scavenger hemoglobin on vasodilating activity of DNIC was established in similar experiments performed in about the same period. In this study, hemoglobin addition to the incubation medium caused an almost complete blockade of the vasodilating effect.

 The ability of hemoglobin to bind NO released spontaneously from mononuclear and binuclear DNIC with thiol-containing ligands was demonstrated recently in direct experiments [32]. The kinetics of binding of NO to hemoglobin was followed spectrophotometrically by absorption at 556 nm characteristic of nitrosyl hemoglobin complexes. This process represents a pseudo-first-order reaction; its limiting step includes spontaneous release of NO from DNIC. Depending on the nature of the thiol-containing ligand, the rate constant for this reaction lies in the range from 1.7 × 10^−3^ to 8.8 × 10^−3^ s^−1^. A Fe^2+^ complex with diethyldithiocarbamate (DETC) localized in hydrophobic compartments of animal tissues was used as an alternative trap of NO released from binuclear DNIC with glutathione [33]. In this case, the air was pumped into animal lungs after preliminary passage through an aqueous solution of DNIC. On entering lung tissues, NO released from DNIC began to bind to Fe^2+^-DETC to yield a mononitrosyl iron-DETC complex. Its characteristic EPR signal (*g*_⊥_ = 2.045 and *g*_||_ = 2.02) had a hyperfine triplet structure at *g*_⊥_; its intensity correlated with NO concentration in animal tissues. Quite expectedly, the most intense EPR signal was recorded in lung tissue, while those recorded in the heart, liver, and kidney were less pronounced [34].

 The release of NO detectable by its vasodilating effect on Roussin's black salt containing dinitrosyl iron fragments was studied in [30, 31]. This effect was significantly enhanced upon irradiation of Roussin's black salt solutions with a laser at 514.2 nm and culminated in decomposition of the salt and release of NO. The NO scavenger hemoglobin and the guanylate cyclase inhibitor methylene blue suppressed the salt-induced vasorelaxation virtually completely.

 These findings suggest that the ability of DNIC with thiol-containing ligands to induce vasodilation (and, as a consequence, to lower arterial pressure in animals and human beings), to suppress platelet aggregation, to accelerate skin wound healing, and to stimulate penile erectile activity in experimental rats is a result of spontaneous release of NO from DNIC. Noteworthily, single i/v injection of M- and B-DNIC able to induce long-lasting hypotension in animals and human patients caused a nearly complete conversion of DNIC into a more stable protein-bound mononuclear paramagnetic form during the transfer of [Fe(NO)_2_] cores of low-molecular DNICs to thiol groups of proteins [27, 35] manifesting a higher (in comparison with low-molecular thiols) affinity for these cores [9]. Protein-bound DNIC acts exclusively as DNIC depots. Most probably, the transfer of NO to its molecular targets is effected by highly mobile low-molecular forms of DNIC whose concentration in cells is determined by the affinity ratio of low-molecular thiols and protein thiol ligands for [Fe(NO)_2_]. An analysis of the shape of the EPR signal of DNIC with thiol-containing ligands localized in animal tissues established that protein-bound M-DNIC make the largest contribution to this signal. As regards low-molecular M-DNIC, their contribution to the total EPR signal of DNIC does not exceed 1% as could be evidenced from the shape of the DNIC EPR recorded in animal tissues at ambient temperature. The protein-bound M-DNIC EPR signal has an anisotropic shape with a halfwidth of 4 mT due to anisotropy of the *g*-factor tensor at *g*_⊥_ = 2.04 and *g*_||_ = 2.014, which correlates with the anisotropy of the EPR signal of low-molecular or protein-bound DNIC with thiol-containing ligands recorded at 77 *K* [9]. Such a coincidence can be attributed to low mobility of the protein globule at ambient temperature, which is insufficient for averaging the anisotropy of the *g*-factor of the EPR signal of protein-bound DNIC. Similar averaging was established in experiments with low-molecular M-DNIC, which manifested high mobility at ambient temperature. Under these conditions, a narrow symmetric signal with a halfwidth of 0.7 mT at *g* = 2.03 was recorded instead of a broad anisotropic EPR signal [9]. Specially designed experiments showed that the appearance of this signal simultaneously with a broad EPR signal of protein-bound DNIC is possible in principle, however, only under conditions where the concentration of low-molecular DNIC is no less than 1% of that of protein-bound DNIC [9]. Since blood levels of protein-bound DNIC, for example, after i/v treatment of rats with 2.5 *μ*moles/kg of low-molecular DNIC, reached 6 *μ*M, the presence of a single broad EPR signal of protein-bound DNIC and the absence of a narrow symmetric EPR signal at *g* = 2.03 characteristic of low-molecular DNIC suggest that blood levels of low-molecular DNIC did not exceed 60 nM [35]. A question arises as to whether the concentration of low-molecular DNIC (60 nM) is too low for manifestation of their hypotensive activity. The clue is in vasodilating effect of these DNIC on isolated blood vessels. DNIC (60 nM) induced more than 50% vasodilation [18], so even very low blood levels of low-molecular DNIC were sufficient for initiating hypotension.

 Let us pass to the consideration of confirming the ability of DNIC with thiol-containing ligands to act as nitrosonium (NO^+^) ion donors. This ability is manifested, in particular, in the S-nitrosating effect of these DNIC on thiols. Here it is appropriate to remember that nitrosonium ions never exist in the free state in aqueous solutions favoring their instantaneous hydrolysis to nitrite ions. Therefore, S-nitrosation takes place only during the transfer of nitrosonium ions from DNIC to thiols and their subsequent direct interaction with NO^+^ ligands under conditions where they remain tightly bound to iron in the course of DNIC decomposition.

 The first evidence in favour of the ability of DNIC with cysteine or glutathione to initiate S-nitrosation of thiols was obtained in our laboratory as early as 1993 [36]. In these studies, the colour of 3 mM solutions of M-DNIC with cysteine changed from green to pink during the first few minutes after a drastic (from 7.4 to 1.0) decrease of pH in air at 60–70°C due to formation of S-nitrosocysteine. Measurements of S-nitrosocysteine content by the intensity of characteristic absorption bands at 340 and 540 nm showed that S-nitrosocysteine concentration was commensurate to that of DNIC (3 mM). However, after the solution acquired its original green colour following drastic elevation of pH from 1.0 to 7.4, the concentration of regenerated DNIC with cysteine dropped down to 50% of the initial level (EPR data). We concluded that at least one nitrosyl ligand in M-DNIC with thiol-containing ligands exists in the form of nitrosonium ions and is responsible for S-nitrosation of cysteine after decomposition of M-DNIC in acid media. The second nitrosyl ligand is released from M-DNIC in the form of a neutral NO molecule. It was also assumed that regeneration of DNIC induced by a rise in pH is controlled by interaction of two S-nitrosocysteine molecules with the iron ion responsible for the 50% decrease of DNIC concentration [36].

 The ability of DNIC with cysteine to S-nitrosate cysteine residues in bovine serum albumin (BSA) was established in previous studies [37]. The level of S-nitrosated BSA was determined from the amount of nitrite (the Griess method) formed upon decomposition of S-nitrosothiol in the presence of mercury ions. In the presence of DNIC with cysteine, S-nitrosated BSA was formed in aqueous solutions at pH 7.4. Addition of 750 *μ*m DNIC with cysteine to 1 mM BSA stimulated the formation of S-nitrosated BSA (85 *μ*m) during 5–8 min. In the presence of an equimolar amount of DNIC with glutathione, the concentration of S-nitrosated BSA formed during the same period reached 40 *μ*m. Oxygen removal had no effect on the yield of S-nitrosated BSA, but further increases in cysteine content strongly enhanced S-nitrosated BSA synthesis.

 S-nitrosation of BSA was established by the biotin method in experiments with M- and B-DNIC with another thiol-containing ligand, namely, thiosulfate (formula ^−^S–S(O_3_)^−^) [38]. Similar transformations induced by mononuclear and binuclear forms of DNIC with thiosulfate were detected for soluble guanylate cyclase from bovine lungs. At maximum (1 and 3 *μ*M, resp.) effective concentrations of M- and B-DNIC, respectively, both forms of DNIC used as NO donors stimulated guanylate cyclase activity to 46.5 and 32.3% of the maximum level attained yet another NO donor, namely, 2,2-diethyl-1-nitroso-oxyhydrazine (DEA/NO) (10 *μ*M). However the increase in the content of M- and B- DNIC to ≥10 *μ*M was accompanied by a complete loss of guanylate cyclase activity. In the presence of 2 mM glutathione, the inhibiting effect of DNIC on the enzyme was eliminated almost completely suggesting that DNIC can induce S-nitrosation of functionally important cysteine residues of guanylate cyclase. Additional evidence in its favor was obtained during estimation of guanylate cyclase S-nitrosation by the biotin method.

 The ability of DNIC with low-molecular thiol-containing ligands to S-nitrosate thiols was studied [39] in the absence and in the presence of *o*-phenanthroline, a widely known potent iron chelator suppressing DNIC synthesis. Cysteine or glutathione solutions (50 mM, 1 mL) were loaded into a Thunberg apparatus (total volume 100 mL) and degassed. Postdegassing treatment with gaseous NO at 100–150 mm Hg stimulated the synthesis of DNIC with cysteine or glutathione (4–6 *μ*m) in the presence of an iron admixture and gradual accumulation of low-molecular S-nitrosocysteine (cys-NO) and S-nitrosoglutathione (GS-NO) in DNIC solutions. At pH 3–5, the yield of both S-nitrosothiols reached 2-3 mM after 5 min. At pH 7–10, the yield of GS-NO was as low as 1 mM, whereas cys-NO synthesis did not take place.

 After injection of a small portion of air into the Thunberg apparatus (until the content of O_2_ (from air) and NO in it reached 10 *μ*moles and 1 mmoles, resp.), the yield of both S-nitrosothiols increased to 10 mM at pH 3–5 and to 5–7 mM at pH 8–10. Noteworthy, after complete dissolution of 10 *μ*moles of O_2_ loaded into the Thunberg apparatus, its concentration in 1 mL of the incubation medium reached 10 mM, that is, was commensurate to the maximum (10 mM) concentration of S-nitrosothiols released from DNIC in the presence of NO and O_2_, respectively. After addition of 0.25 mM *o*-phenanthroline prior to its interaction with NO or NO + O_2_, the synthesis of S-nitrosothiols was completely blocked both in the presence and in the absence of oxygen. This blockade correlated with inhibition of synthesis of DNIC with thiol-containing ligands (cysteine or glutathione) detected by a characteristic EPR signal in the absence of *o*-phenanthroline. This suggests that it is these DNIC that play the role of catalysts triggering S-nitrosation of *∼*20% of thiols present in the solution [39].

 Based on these findings, we assumed that augmented synthesis of S-nitrosothiols in air is a result of destabilizing effect of oxygen on DNIC as can be evidenced from the disappearance of the EPR signal at *g* = 2.03. This hypothesis was corroborated by experiments in which 1 mM solutions of BSA and equine methemoglobin were loaded into the Thunberg apparatus in a volume of 1 mL. Further addition of DNIC with phosphate (1 mM) was accompanied by the formation of protein-bound DNIC. The latter disappeared after loading of the NO + O_2_ mixture to the Thunberg apparatus, but an absorption band characteristic of nitrosated BSA and equine methemoglobin (~1 mM) appeared in the range from 340 to 360 nm. Noteworthy, treatment of proteins with the NO + O_2_ mixture in the absence of DNIC was not accompanied by generation of the nitrosated form of the proteins. However, addition of Fe^2+^ and cysteine to nitrosated proteins initiated the synthesis of low-molecular DNIC with cysteine as a result of which the band corresponding to nitrosated protein groups (cysteine and tryptophan residues) disappeared from their optical absorption spectra. Speaking differently, after transfer of their nitrosyl components to iron and thiol, nitrosated protein fragments provoked the synthesis of DNIC [39]. As in the previous study [39], the conclusion to the crucial role of DNIC in initiating the synthesis of S-nitrosothiols in cultured RAW264.7 cells was made on the basis of the ability of the iron chelator salicylaldehyde isonicotinoyl hydrazone (SIH) to penetrate deeply inside cells and to suppress DNIC synthesis in the presence of exogenous NO with subsequent formation of S-nitrosothiols [40]. It is direct correlation between intracellular concentrations of DNIC and S-nitrosothiols that led the authors to suggest that S-nitrosation of thiols in cultured cells is controlled by their interaction with DNIC responsible for the transfer of nitrosonium ions to thiol groups of proteins or low-molecular thiols. This hypothesis is consistent with the results of the authors according to which the synthesis of S-nitrosothiols is only weakly or not at all dependent on oxygen content in the intracellular space because, similar to DNIC, S-nitrosothiols appear in cells even at very low (<8 *μ*M) concentrations of O_2_, which calls into question the current viewpoint on the crucial role of higher NO oxides formed upon oxidation of NO by oxygen in the course of S-nitrosation of thiols in cells and tissues [40].

 Augmented synthesis of S-nitrosothiols in NO-treated yeast cells in the absence of oxygen was described in [41]. Addition of a strong Fe^2+^ chelator 1,10-phenanthroline to a yeast-containing incubation medium prior to NO treatment strongly inhibited the synthesis of low-molecular and, particularly, protein-bound S-nitrosothiols. This finding led the authors to conclude that a prominent role in this event belongs to DNIC as a donor of nitrosonium ions whose binding to thiols gives corresponding S-nitrosothiols.

 An overview of the published data suggests that DNIC with thiol-containing ligands can indeed S-nitrosate thiols, most probably, due to the presence of NO^+^ ions as ligands. Two questions arise as to how treatment of aqueous solutions of Fe^2+^ and thiols with gaseous NO triggers the conversion of NO into NO^+^ in the course of DNIC synthesis and why binding of nitrosonium ions to DNIC prevents their hydrolysis under conditions where free nitrosonium ions undergo fast hydrolysis in aqueous solutions to be further converted into nitrite anions.

 A clue to these questions may come from the analysis of mechanisms of formation of DNIC and their electronic and spatial structures. It was stated earlier in this chapter that the simplest way to obtain paramagnetic M-DNIC with thiol- containing ligands is treatment of Fe^2+^+ thiol-containing aqueous solutions with gaseous NO at pH 7–7.5, the latter being taken in excess [16, 17]. In this system, binding of two NO molecules to Fe^2+^ favors the formation of the DNIC-Fe(NO)_2_ core. However, because of the presence in the latter of an even number of unpaired electrons (six d-electrons and two unpaired electrons within the composition of NO ligands) it can exist either in the diamagnetic (*S* = 0), or in the paramagnetic high-spin states characterized with a whole value of the spin. This generates a new question: what is the mechanism whereby DNIC pass into the paramagnetic state with *S* = 1/2 where they generate an EPR signal that can be recorded at ambient temperature and is more characteristic of DNIC with thiol-containing ligands?

 Previous studies showed that the synthesis of DNIC with non-thiol ligands is accompanied by the formation of nitrous oxide (N_2_O) [42]. This finding prompted us to suggest a hypothetical mechanism of M-DNIC formation, of N_2_O formation, in particular [39, 43]. We proceeded from the assumption that binding of two NO molecules to Fe^2+^ ions results in their disproportionation and conversion into nitrosonium and nitroxyl ions (NO^+^ and NO^−^, resp.). Protonation of the latter yields nitroxyl (HNO), which leaves the coordination sphere of Fe^2+^. Recombination of two nitroxyl molecules gives nitrous oxide (Scheme 1); subsequent incorporation of the NO molecule into M-DNIC instead of nitroxyl is accompanied by a transfer of the unpaired electron from the latter to iron and formation of the {Fe^+^(NO^+^)_2_} fragment and, as a consequence, of M-DNIC with thiol-containing (RS^−^) ligands of the formula {(RS^−^)_2_Fe^+^(NO^+^)_2_}^+^ with a d^7^configuration of the iron atom ({Fe(NO)_2_}^7^ according to the Enemark-Feltham classification [44]). (For the presence in DNIC of only two thiol-containing ligands see below). The concomitant formation of nitrous oxide (N_2_O) in the course of M-DNIC synthesis and the involvement of three NO molecules in the formation of one M-DNIC were confirmed in more recent studies [45, 46].

 It is of note that disproportionation of NO molecules in the gas phase described by the equation 3 NO = N_2_O + NO_2_ [47] proceeds at a high rate and at high (≥10 atm) NO pressure, which leads to fast accumulation of NO_2_, for example in pressure gas bottles initially containing “pure” NO. This creates a handicap to the use of bottled gaseous NO in experiments with NO addition to biosystems: removal of NO_2_ from the solutions demands additional passage of bottled gas through strongly alkaline solutions. At low (<1 atm) NO pressures, the collision rate of NO molecules stimulating their disproportionation decreases. As a result, disproportionation of free NO molecules does cannot play any significant role in the formation of DNIC with thiol-containing ligands. However, after binding of these two molecules to Fe^2^ (Scheme 1) the probability of this reaction can increase dramatically even at low concentrations of NO in the solution. The increase in the electron density on one of NO molecules and its corresponding decrease on the second NO ligand proceeds through the formation of molecular orbitals (MOs) including NO orbitals and d-orbitals of iron. Formally, this corresponds to electron transfer between two NO molecules and favors disproportionation between two NO molecules bound to Fe^2+^.

 The first step in this process (see Scheme 1), namely, formation of  low-spin mononitrosyl iron complexes (MNICs) with thiol-containing ligands, is omitted. It was found [48, 49] that MNICs are generated during interaction of Fe^2+^ and thiols with NO whose concentration in the solution is of the same order as those of iron and thiols. With an increase in NO content in solutions of MNIC with thiol-containing ligands and the involvement of the second NO molecule into MNIC the latter are converted into corresponding DNIC. Similar to DNIC, MNIC with thiol-containing ligands exist in a low-spin state (*S* = 1/2), which facilitates the registration of their EPR signals at ambient temperature, where the signal has a shape of a symmetric triplet at *g* = 2.04 with splitting at 1.2 mT due to hyperfine interaction of the unpaired electron with the nitrogen core of the nitrosyl ligand [48, 49].

 Our hypothetical mechanism of low-spin M-DNIC synthesis has two obvious arguments in its favour. First, it provides an explanation for the transition of the Fe^2+^(NO)_2_ fragment into the paramagnetic state initially characterized by an even number of electrons acquiring a diamagnetic configuration upon pairing. Second, it sheds additional light on the origin of nitrosonium ions as DNIC ligands.

 The results of analysis of EPR signals of M-DNIC with thiol-containing ligands [4, 8, 9, 15, 16, 50, 51] (Figure 2) are in good agreement with our hypothetical mechanism of formation, composition, and distribution of unpaired electron density in these complexes. First, according to EPR data in the presence of hyperfine structure (HFS) components water-soluble M-DNIC with thiol-containing ligands generate symmetric EPR signals with narrow (0.08–0.15 mT) components at ambient temperature and anisotropic signals with easily resolvable components at *g*_⊥_ and *g*_||_ at 77 K [8–10, 15, 16, 50, 51] suggesting their low-spin (*S* = 1/2)d^7^ configuration [16, 50–52]. Second, the high value of hyperfine splitting in doublet HFS from the ^57^Fe nucleus (1.25 mT) incorporated into M-DNIC (Figure 2(d, right side)) is explicitly suggestive of predominant localization of unpaired electron density on the iron atom, that is, is fully consistent with the hypothetical structure and mechanism of synthesis of these DNIC. Third, the low level of hyperfine splitting in quintet HFS (0.15 mT) characteristic of EPR signals of DNIC with mercaptotriazole (Figure 2(c, right side)) during interaction of unpaired electron density with nitrogen nucleus of nitrosyl ligands (<0.3% of the maximum level). This corresponds to the ionized (NO^+^) state of nitrosyl ligands into which these ligands are transformed in the course of M-DNIC synthesis (Scheme 1). Moreover, the quintet origin of HFS points unambiguously to identity of both nitrosyl ligands in DNIC. Fourth, additional evidence for the presence of two nitrosyl and two thiol ligands in DNIC with cysteine or glutathione can be derived from the analysis of a hyperfine 13-component structure (HFS) characteristic of narrow EPR signals recorded for these DNIC at ambient temperature (*g* = 2.03) (Figure 2(a, right side)). This HFS is formed upon interaction of the unpaired electron with two nitrogen nuclei (^14^N, nuclear spin *I* = 1, triplet HFS from each nucleus) and four methylene group protons (*I* = 1/2, doublet HFS from each proton) in two nitrosyl and two cysteine ligands, respectively. After substitution of ^14 ^N for ^15 ^N (*I* = 1/2, doublet HFS from each nucleus) in nitrosyl ligands, the total number of HFS components decreases to 9 (Figure 2(b, right side)). The low (0.15 and 0.07 mT) level of hyperfine splitting on N nuclei and protons suggests that the negligible amount of unpaired electron density is localized on both nitrosyl and thiol ligands in DNIC with glutathione or cysteine ligands. This finding suggests that in these DNIC nitrosyl ligands also exist in the ionized (NO^+^) state (Scheme 1).

 It would be natural to expect that fast hydrolysis of NO^+^ ligands might initiate fast decomposition of M-DNIC. Indeed, storage of M-DNIC solutions is accompanied by gradual disappearance of M-DNIC within 10 or more minutes. However, this is unrelated to decomposition of their Fe(NO)_2_ core, but, rather, to conversion of M-DNIC into a more stable binuclear form (B-DNIC), the Fe(NO)_2_ cores in which retain their integrity for several hours and even several days. Addition of thiols to B-DNIC solutions results in reassembly of M-DNIC to an extent where their concentration reaches 50% of the initial concentration [15].

 What is the reason for such remarkably high stability of DNIC with thiol-containing ligands against the background of NO conversion into nitrosonium ions (Scheme 1)? In our opinion, the above-cited formula of M-DNIC reflects exclusively the distribution of the bulk of the spin density (suggestive of predominant localization of the unpaired electron on the iron atom and its insignificant distribution on nitrosyl and thiol ligands). At the same time, the formation of molecular orbitals of DNIC including electron d-orbitals of iron, sulfur atoms, and nitrosyl ligands is accompanied by significant redistribution of the electron charge in DNIC, most probably, as a result of transfer of electron density from sulfur atoms to iron atoms and further to nitrosyl ligands. Under these conditions, the positive charge of the nitrosyl ligands diminishes appreciably, the interaction of the ligands with hydroxyl ions weakens, and, as a consequence, hydrolysis of nitrosyl ligands does not take place.

 A natural question arises: what is the mechanism whereby NO^+^ ligands in DNIC with thiol-containing ligands induce S-nitrosation of thiols? In all probability, their S-nitrosating activity is a result of weak binding of thiol-containing ligands to iron molecules under conditions favoring destabilization of DNIC. The disappearance of electron density from nitrosyl ligands increases their positive charge and strongly enhances their S-nitrosating activity. Noteworthy, nitrosonium ions do not interfere with their binding to iron up to the moment of their transfer to thiol groups of proteins or low-molecular compounds. In other words, nitrosonium ions can hardly manifest any S-nitrosating activity after transition to the free state where they undergo fast hydrolysis to nitrite ions.

 An analysis of EPR signals of DNIC with thiol-containing ligands not only allowed us to determine their composition and distribution of unpaired electron density in them, but also to establish the predominant localization of the unpaired electron on the d-orbital from the ratios of the components of the *g*-factor and the *A*(^57^Fe) tensor. Moreover, it made it possible to detect and specify the localization of d-orbitals, or, more precisely, of molecular orbitals (MOs) the main contribution to which is made by d-orbitals of iron and to propose a hypothetical spatial structure of DNIC. All these questions can be solved through the analysis of the equations for the *g*-factor and the HFS tensors from the Co^2+^ nucleus for low-spin (*S* = 1/2) complex of Co^2+^ with phthalocyanine or porphyrin ligands described in [53]. The complex is characterized by a d^7^ configuration of the central atom and effective D_4h_ symmetry (in some instances). Similar characteristics are displayed by M-DNIC with thiol-containing ligands: the hypothesis on the effective D_4h_ symmetry of these DNIC [50] relies on the presence of only two distinct values of the *g*-tensor components for DNIC (*g*_⊥_ and *g*_||_). It seems therefore well grounded to use the equations [53] to estimation of the components of the *g*-factor and the *A*(^57^Fe) tensor characteristic of DNIC with thiol-containing ligands.

 The experimental values of the g-factor and the *A*(^57^Fe) tensors for EPR signals of DNIC with thiol-containing ligands are as follows: *g*_⊥_ = 2.040, *g*_||_ = 2.014 (*g*_aver._ = 2.03), *A*_⊥_(^57^Fe) = −1.7 mT, *A*_||_(^57^Fe) = −0.25 mT, *A*_iso_ = −1.22 mT (DNIC with cysteine) [8, 50, 51, 54]; *g*_⊥_ = 2.042, *g*_||_ = 2.014 (*g*_aver._ = 2.033), *A*_⊥_ (^57^Fe) = −1.7 mT, *A*_||_(^57^Fe) = −0.35 mT, *A*_iso_ = −1.25 mT (DNIC with ethyl xanthogenate) [51, 55]; *g*_⊥_ = 2.045, *g*_||_ = 2.014 (*g*_aver._ = 2.035), A_⊥_(^57^Fe) = −1.7 mT, *A*_||_(^57^Fe) = −0.3 mT, *A*_iso_ = −1.25 mT (DNIC with thiosulfate) [51]. For all these cases, *g*_⊥_ > *g*_||_ > 2.0023 represents purely spin values of the *g*-factor. Such ratio of *g*-factor values is consistent with unpaired electron localization on d_*z*_^2^ iron orbital for low spin d^7^configuration (*S* = 1/2) and square planar spatial structure [50] (or elongated octahedra containing weak ligands along the *z* axis: molecules H_2_O or any other solvents). Similar conclusion was made for low spin d^7^ [Fe(CN)_4_Cl_2_]^5−^ complex with localization of the unpaired electron on d_*z*_^2^ orbital, characterized with elongated octahedral structure [56]. The description of electron and spatial structures of DNIC in detail is given below after the analysis of the ratio of *g*- and *A*(^57^Fe) tensor values.

 From the above-mentioned experimental data, the ratio of the module of values of the ^57^Fe-HFS tensor appears as:

(1) |A⊥(Fe57)|>|A||(Fe57)|.

From known experimental values of *A*_⊥_(^57^Fe) and *A*_||_(^57^Fe), the values of *P*  (*P* = 2.0023*g*_*N*_ *β*_*e*_ *β*_*N*_1/*r* ^3^>) and *K* (Fermi's contact interaction constant) can be determined using a system of equations [53] as +1.9 mT (+18 × 10^−4^ cm^−1^) and −1.25 mT (−12 × 10^−4^ cm^−1^), respectively. These values will be used further in this paper.

 Let us pass to the consideration of the ratios of the components of the *g*-factor and the ^57^Fe-HFS tensor derived from equations [53] with respect to the d-orbitals on which the unpaired electron is localized. From the equations it follows that for the d^7^configuration *g*_⊥_ > *g*_||_ > 2.0023, if the unpaired electron is localized on the d_*z*2_ orbital, but *g*_⊥_ > *g*_||_ (NB: *g*_||_ < 2.0023), if the unpaired electron is localized on the d_*x*2−*y*2_ or on the d_*xy*_ orbital for any other type of the coordinate system. If the unpaired electron is localized on the d_*yz*_ or d_*xz*_ orbital, *g*_*y*_ < *g*_*z*_ and *g*_*x*_ < *g*_*z*_, respectively. Stipulating that *g*_⊥_ > *g*_||_ > 2.0023 for the M-DNIC EPR signal, the unpaired electron in them will be explicitly localized on the d_*z*2_ orbital.

 A similar conclusion can be made from the analysis of experimental values of the *A*(^57^Fe) tensor. Considering that the maximum deviation of the *g*-factor from the purely spin value is <0.05, the values of *c*_*i*_ and *c*_*i*_^2^ reflecting the discrepancies between the equations [53] can also be insignificant suggesting that the latter make a negligibly small contribution to the equations for components of the *A*(^57^Fe) tensor [53]. As a result the equations include only *K* and components of the tensor for the dipole-dipole interaction of the electron spins with the core (hereinafter designated as *A* ^*D*^). Hence,

(2) Ai(Fe57)=K  +  AiD.

Substituting the components of the *A*_*i*_^*D*^ tensor into (2) (using the values of the *A*_*i*_^*D*^ tensor for localization of the unpaired electron on different d-orbitals [57]), for (*K* < 0) we obtain: |*A*_*z*_| < |*A*_*x*_| = |*A*_*y*_| for the d_*z*2_ orbital (|*K*| > 1/7*P*); |*A*_*z*_| > |*A*_*x*_| = |*A*_*y*_| for the d_*x*2−*y*2_ (d_*xy*_) orbital; |*A*_*z*_| = |*A*_*y*_| < |*A*_*x*_| for the d_*yz*_ orbital; |*A*_*z*_| = |*A*_*x*_| < |*A*_*y*_| for the d_*xz*_ orbital. Judging from these ratios and stipulating that for DNIC |*A*_*x*_| = |*A*_*y*_| = |*A*_⊥_|(1.7 mT) > |*A*_*z*_||*A*_||_|(0.3 mT) and |*K*| > 1/7*P* (*K* = −1, 25 mT and *P* = +1, 9 mT), the unpaired electron is unambiguously localized on the d_*z*2_ orbital.

 This finding is fully consistent with the antibonding MO diagram (where the main contribution is made by d-orbitals of iron) of DNIC with thiol-containing ligands having a d^7^ low spin configuration with localization of the unpaired electron on d_*z*_^2^ orbital and a square planar spatial structure proposed in [50]. High energy of MO(d_*x*2−*y*2_) is provided by strong interaction of the d_*x*2−*y*2_ orbital with orbitals of two thiol and two nitrosyl ligands in the *xy* plane. The interaction of the d_*z*2_ orbital with these ligands is also accompanied by notable increment of MO(d_*z*2_) energy, which is still much less pronounced compared to MO(d_*x*2−*y*2_). Further increases in MO(d_*z*2_) energy can be due to weak interactions of the *d*_*z*2_ orbital with molecules of H_2_O or any other solvent: these weak ligands can occupy two vacant apical positions along the *z* axis of DNIC. At the same time, interaction of d_*xy*_, d_*xz*_, and d_*yz*_ orbitals (t_2_-manifold) with corresponding combinations of the NOπ* orbitals are expected to decrease the energy of MO(d_*xy*_), MO(d_*xz*_), and MO(d_*yz*_). Under these conditions, MO(NOπ*) (where the major contribution is made by NOπ* orbitals) can be localized between MO(d_*x*2−*y*2_) and MO(d_*z*2_).

 As mentioned earlier with reference to EPR data, DNIC contain four strong ligands (two NO^+^and two RS^−^ ligands). Stipulating that frozen solutions of DNIC with a bidentate thiol-containing ligand, for example, ethyl xanthogenate, generate EPR signals coinciding virtually completely with EPR signals of DNIC with other thiol-containing ligands [55], we hypothesized that thiol-containing (and, correspondingly, nitrosyl) ligands in these DNIC are in the *cis*-position [50].

 As stated earlier in this chapter, the hypothetical structure of {(RS^–^)_2_Fe^+^(NO^+^)_2_}^+^ complexes reflects the distribution of spin density within them. High *π*-donor activity of sulfur atoms in thiol-containing ligands initiates a transfer of electron density (part of paired electrons) from sulfur atoms to iron and NO^+^ ligands as a result of which their positive charge and hydrolytic effect of hydroxyl ions diminish, which, may result in stabilization of DNIC the nitrosyl ligands in which are formally represented by nitrosonium ions.

 The above hypotheses on electronic and spatial structures of DNIC with thiol-containing ligands correlate, in many features, with the large body of evidence on corresponding characteristics of low-spin mononitrosyl iron complexes (MNIC) with thiol-containing or other strong ligands, for example, cyanide. In terms of the Enemark-Feltham classification, the core structure of low-spin MNIC is described as Fe^+^(NO^+^), that is, is similar to that of DNIC (Fe^+^(NO^+^)_2_). In both cases, these structures reflect the distribution of spin density on these cores as corresponding to localization of the unpaired electron on the iron atom (d^7^) and a lack of unpaired electrons on nitrosyl ligands. However, this description is purely formal and does not reflect the true distribution of electron density and the actual charge on these cores [58]. Actually, the extremely strong d-π* back-donation characteristic of MNIC increases the electron density on “empty” NOπ* orbitals [58, 59]. This phenomenon is enhanced in the presence, in MNIC, of thiol-containing ligands manifesting high electron-donor activity and promoting the transfer of electronic density to the Fe^+^(NO^+^) core [60].

 A prominent feature of the electronic structure of MNIC and DNIC is the presence in NO ligands of two low-energy NOπ* orbitals. This indicates that the design of MO diagrams for these complexes should take into consideration the crucial contribution of both d- and NOπ* orbitals to respective MO. The MO(NOπ*) are localized between first-type MOs. By illustration, let us consider the results of analysis of MO diagrams for nitroprusside [Fe(CN)_5_NO]^2−^, namely, MNIC with cyanide ligands. After acceptance of one electron, nitroprusside passes into the paramagnetic state [Fe(CN)_5_NO]^3−^ [61, 62] or [Fe(CN)_4_NO]^2−^ characterized by the loss of one cyanide ligand initially localized in the *trans*-position relative to NO [63]. Because of strong interaction of iron with cyanide ligands and NO in the *xy* plane and along axis *z*, MO(d_*x*2−*y*2_) and MO(d_*z*2_) occupy a much higher position in the reduced complex [Fe(CN)_5_NO]^3−^, than MO(NOπ*), the degeneracy of MO(NOπ*) being removed in the complex. As a result, the unpaired electron in the low-spin (*S* = 1/2) complex is localized on one of MO(NOπ*) [61, 62]. As regards the [Fe(CN)_4_NO]^2−^ complexes, the energy of the MO(d_*z*2_) orbital in them is decreased because of the lack of the cyanide ligand in the axial position. as a result, the unpaired electron moves from MO(NOπ*) to MO(d_*z*2_) in such a way that the unpaired electron becomes predominantly localized on the d_*z*2_ orbital [51, 64]. Supporting evidence for such localization can be derived from characteristics of their EPR signal: *g*_⊥_ = 2.032, *g*_||_ = 2.005, *g*_aver._ = 2.023  (*g*_⊥_ > *g*_||_ > 2.0023), |*A*_⊥_(^57^Fe)| > |*A*_||_(^57^Fe)| [62], resp.) consistent with localization of the unpaired electron on the d_*z*2_ orbital [51, 62, 64].

 Similar characteristics of the EPR signal were established for low-spin MNIC with thiol-containing ligands [52, 55, 65]. Thus, for MNIC with bidentate thiol-containing compounds (xanthogenate and dithiocarbamate), the values of the g-factor of the EPR signal are as follows: *g*_⊥_ = 2.044, *g*_||_ = 2.025, (*g*_aver._ = 2.038) and *g*_*x*_ = 2.039, *g*_*y*_ = 2.035, *g*_*z*_ = 2.025  (*g*_aver._ = 2.033), respectively [52, 65]. The values of A(^57^Fe) for MNIC with dithiocarbamate were determined as |*A*_⊥_(^57^Fe)| > |*A*_||_(^57^Fe)| [65]. This value is also consistent with localization of the unpaired electron on d_*z*2_ orbitals of both complexes [52, 55, 65]. In the framework of the third-order perturbation theory, the difference between the values of *g*_||_ and *g*_*z*_ and that of the purely spin *g*-factor (2.0023) can be attributed to the interference of low-quartet states in the ground doublet state of the complexes [53].

 Thus, low-spin MNIC and M-DNIC can be integrated into a single group of nitrosyl iron complexes having d^7^ electronic configuration with predominant localization of the unpaired electron on MO(d_*z*2_) and D_4h_ or C_2v_ symmetry.

 The presence in M-DNIC of two nitrosyl and two sulfur-containing ligands and the tetrahedral spatial structure of these complexes in the crystal state were confirmed by X-ray diffraction analysis data [54, 66–68]. However, the tetrahedral field of these ligands cannot induce pairing of the spins in the d^7^configuration to the low-spin (*S* = 1/2) state even under conditions of its strong distortion [54]. As a result, tetrahedral DNIC complexes having a d^7^ configuration exist in the high-spin state (*S* = 3/2). Therefore, many investigators concerned with the study of DNIC with thiol-containing ligands share an opinion that according to Enemark-Feltham classification the latter should be described by the formula {Fe(NO)_2_}^9^, that is, localization of nine electrons on five MO(d) in tetrahedral DNIC complexes is responsible for the low-spin (*S* = 1/2) state of M-DNIC. Two alternative models of M-DNIC have been developed according to which the low-spin (*S* = 1/2) state of M-DNIC is determined by antiferromagnetic interaction between the iron atom and two nitrosyl ligands. In the framework of the first model, the structure of the DNIC core appears as [Fe^3+^(NO^−^)_2_] (or as {Fe(NO)_2_}^9^ according to Enemark-Feltham classification) and has a high-spin (*S* = 5/2) d^5^-configuration of the iron atom and a triplet (*S* = 1)  state of nitroxyl ligands [68, 69]. According to the second model, DNIC with a core structure [Fe^+^ (NO)_2_] {Fe(NO)_2_}^9^ (in terms of Enemark-Feltham's classification) is characterized by a high-spin (S = 3/2) d^7^-configuration of the iron atom bound to two neutral NO molecules (*S* = 1/2) [66, 67]. Both models have a tetrahedral spatial structure.

 Here, it is necessary to note that by postulating the {Fe(NO)_2_}^9^ structure for DNIC with thiol-containing ligands [66–69] the authors did not go into detail about the mechanism of formation of a paramagnetic DNIC core with an odd number of electrons in the low-spin (*S* = 1/2) state. Both core structures, that is, [Fe^3+^(NO^−^)_2_] and [Fe^+^(NO)_2_], were postulated for M-DNIC prepared by substitution of carbonyl ligands for thiolate compounds into appropriate ready-to-use DNIC with carbonyl and nitrosyl ligands. Moreover, it was assumed that the corresponding {Fe(NO)_2_}^9^ structure characteristic of DNIC with thiol-containing ligands in the crystal state and the tetrahedral spatial structure of DNIC are preserved after dissolution of DNIC crystals in a appropriate solvent. In this case we deal with a complete variance between our model of electron and spatial structures of soluble DNIC with thiol-containing ligands and the models described in [66–69]. A question arises, which of these models reflecting the structure and physico-chemical characteristics of soluble DNIC is adequate and genuine? A comprehensive analysis showed that our viewpoint is more correct. 

 First, our model provides a natural interpretation for the ability of DNIC with thiol-containing ligands to induce S-nitrosation of thiols due to the presence of nitrosonium ions (NO^+^) in the DNIC [Fe^+^(NO^+^)_2_] core. As mentioned above, this formula reflects the distribution of spin electron density within the core. Actually, the electron density (i.e., part of paired electrons) is transferred from sulfur atoms to iron and NO^+^ ligands due to high *π*-donor activity of sulfur atoms of thiol-containing ligands thereby decreasing their positive charge and attenuating the hydrolytic effect of hydroxyl ions. The ability of nitrosyl ligands to induce S-nitrosation of thiols diminishes, correspondingly, and reappears upon decomposition of DNIC or after attaining a chemical equilibrium between DNIC and its constituent components (Scheme 2), that is, during the release of thiol-containing ligands from DNIC. As a result, the electron density on nitrosyl ligands gradually disappears as a result of which the latter are converted into nitrosonium ions and thus acquire a positive charge. Only one nitrosyl ligand remains in the form of NO^+^, whereas the second ligand accepts an electron from iron to be converted into a neutral NO molecule. This mechanism underlies the ability of DNIC to act as NO and NO^+^ donors.

 The ability of these DNIC to induce S-nitrosation of thiols can hardly be explained in terms of the {Fe(NO)_2_}^9^ structure of DNIC. According to two alternative formulas for the DNIC-[Fe^3+^(NO^−^)_2_] and [Fe^+^(NO)_2_] cores [66–69], these DNIC donate only NO molecules. In the former case, the release of NO is provoked by a transfer of the unpaired electron from the nitroxyl ion to the iron atom; in the latter case, this does not take place. Therefore, the authors postulating the {Fe(NO)_2_}^9^ structure of DNIC in both crystal and soluble states insistently suggest that the ability of DNIC to S-nitrosate thiols is determined by NO molecules liberated from DNIC. This suggests that S-nitrosation can be provoked indirectly, for example, through formation of nitrogen dioxide in the course of oxidation of NO molecules released from DNIC. According to present-day concepts, nitrogen dioxide possesses an ability to S-nitrosate thiols when used as a N_2_O_4_ dimer or N_2_O_3_ after its binding to NO [70].

 This hypothesis was first stated in experiments illustrating the ability of M-DNIC with cysteine for *in vivo* S-nitrosation of intracellular proteins in EA.hy926 cells obtained by fusion of primary human umbilical vein endothelial cells with human lung carcinoma cells [71]. The concentration of S-nitrosated proteins determined by the biotin method increased after incubation of cells with DNIC, reaching the maximum level after 24 h. The {Fe(NO)_2_}^9^ structure of M-DNIC (note: the structure of the core complex appears as [Fe^+^(NO)_2_]) led the authors to conclude that S-nitrosating activity of DNIC with cysteine is determined by NO released from DNIC and its subsequent oxidation to nitrogen dioxide.

 This hypothesis is in conflict with the results of other authors (Lancaster's group [40] and Stamler's group [41]) who succeeded in demonstrating that DNIC S-nitrosate thiols in body cells with equal efficiency in the presence and in the absence of oxygen. Therefore, the presence, in DNIC, of nitrosonium ions seems to be the only factor responsible for S-nitrosation suggesting that it is only {Fe(NO)_2_}^7^ DNIC with the core structure [Fe^+^(NO^+^)_2_]) that initiate this process.

 Second, the formation of [Fe^3+^(NO^−^)_2_] or [Fe^+^(NO)_2_] cores in M-DNIC with thiol-containing ligands is difficult to explain in the paradigm of the simplest procedure used for the synthesis of M-DNIC formed upon treatment of Fe^2+^ + thiol solutions with gaseous NO [16, 17]. As regards our hypothetical {Fe(NO)_2_}^7^ structure of M-DNIC, the formation of the [Fe^+^(NO^+^)_2_] core in M-DNIC can be easily explained in the framework of the mechanism depicted in Scheme 1. The concomitant formation of nitrous oxide (N_2_O) in the course of M-DNIC synthesis and the involvement of three NO molecules in the formation of one M-DNIC were confirmed in experiments carried out by other authors [45, 46].

 Third, by proposing a formula for this M-DNIC core as [Fe^3+^(NO^−^)_2_] or [Fe^+^(NO)_2_] in the framework of the {Fe(NO)_2_}^9^ structure, the authors neglect the significant effects of anionic thiol- and non-thiol-containing ligands in DNIC on their electronic and spatial structures. At the same time, substitution of thiol-containing ligands for, for example, phosphate initiates significant alteration of their electronic structures detectable by changes in EPR characteristics of DNIC [54, 72]. In contrast to DNICs with thiol-containing ligands, which exist exclusively in the low-spin state, nitrosyl iron complexes with non-thiol (phosphate, citrate, ascorbate, etc.) ligands can exist in two forms, namely, as low-spin M-DNIC (S = 1/2) form (*g*_aver._ = 2.03–2.04, halfwidth −5–6 mT) and as a high-spin mononitrosyl iron complex (MNIC) (*S* = 3/2) with an intensive peak on the broad EPR signal at *g* = 4.0 [54, 72]. MNIC with phosphate contain the bulk of incorporated iron and retain their stability within at least one hour. As regards low-spin M-DNIC with phosphate, they are completely decomposed within the first 0–15 min after removal of gaseous NO from the solution in which their synthesis is performed as can be evidenced from the appearance of nitrite detectable by HPLC. Presumably, their decomposition is induced by hydrolysis of nitrosonium ions within the composition of these DNIC (unpublished data).

 Fourth, our hypothesis on the structure of {Fe(NO)_2_}^7^ M-DNIC sheds additional light on some of their EPR characteristics, namely, ratios of the components of the *g*-factor and *A*(^57^Fe) tensors characteristic of low-spin M-DNIC with d^7^ electron configuration and an unpaired electron localized on MO(d_*z*2_). The correspondence of EPR characteristics of soluble DNIC to their chemical formulas ([Fe^3+^(NO^−^)_2_] or [Fe^+^(NO)_2_]) did not receive proper attention of the authors of the aforecited formulas either. In the meantime, the equation proposed in [73] can be used for estimating the values of the *g*-factor tensor of the EPR signal of DNIC, namely, the one having a [Fe^+^(NO)_2_] core. According to the authors, the low-spin (*S* = 1/2) state of this core is provided by the antiferromagnetic interaction of Fe^+^ with a high-spin (*S* = 3/2) d^7^configuration and nitrosyl NO ligands (S = 1/2) with the overall spin *S* = 1 [67, 68]. Such a spin-paired system can be regarded as a mixed triad of interchanging magnetic centers, namely, two identical M* (two NO molecules) and one M^#^ (iron ion) (provided the exchange interaction is ≫ of the quantum energy of the microwave electromagnetic field of the EPR radiospectrometer), and the value of the *g*-factor for this triad cluster described by the following equation [73] can be calculated as:

(3) $$\;\begin{array}{c} g=g_{3}+\frac{\left(g_{3}-g_{i}\right)\left[S_{3}\left(S_{3}+1\right)-S_{1,2}\left(S_{1,2}+1\right)-S\left(S+1\right)\right]}{2S\left(S+1\right)}, \end{array}$$

where *g*_*i*_  (*g*_*i*_ = *g*_1_ = *g*_2_) is the g-factor tensor of the EPR signal characteristic of both identical magnetic centers of M* (two NO molecules), *S*_1,2_ is the total spin of these centers equal to unity, *g*_3_ is the g-tensor and *S*_3_ is the spin of the magnetic center M^#^ (iron ion) equal to 3/2 and S is the total spin of triad cluster equal to 1/2. By substituting the values of the corresponding spins into (3), we obtain

(4) $$g=\;g_{3}+\frac{2}{3}\left(g_{3}-g_{i}\right)=\frac{5}{3}g_{3}-\frac{2}{3}g_{i}.\;$$

Since the value of the g-tensor (*g*_3_) for the iron ion in the high-spin (*S* = 3/2) state is equal to *g*_⊥_ ~ 4 and *g*_||_ ~ 2 [74] and both values of the g-tensor (*g*_*i*_) for bound NO (*S* = 1/2) are approximately equal to 2 [75], by substituting these value into (4) we obtain the value of the *g*_⊥_ component of the *g*-factor tensor of the cluster equal to 5.3. This value differs drastically from the experimentally determined value of *g*_⊥_ for the EPR signal of DNIC with thiol-containing ligands (*g*_⊥_ = 2.04), that is, this model of DNIC core is inadequate to the genuine structure of these DNIC.

 Let us consider now the model of DNIC with a configuration of {Fe(NO)_2_}^9^ and a [Fe^3+^(NO^−^)_2_] core [66, 68, 69]. Their low-spin (*S* = 1/2) state is provided by antiferromagnetic interaction between iron in the high-spin (*S* = 5/2) state and a d^5^ electronic configuration and nitrosyl ligands of NO^−^ in the triplet state (*S* = 1). As (3) was derived during the analysis of magnetic systems with a half/whole value of the spin [73], it will not be used for the analysis of the model of the DNIC core of [Fe^3+^(NO^−^)_2_]. However, the inconsistency of this model to the genuine structure of DNIC becomes especially apparent, if one takes into consideration the fact that DNIC with thiol-containing ligands are characterized by weak spin-lattice interactions as a result of which only narrow components of their EPR signal (0.08 mT) can be recorded at ambient temperature [8, 15, 49, 51, 54]. With a decrease in the registration temperature to 77 K, used for recording the anisotropic EPR signal, the width of the spin-packet does not exceed 0.1 mT [2]. At the same time, systems with exchange magnetic interactions (including both hypothetical models of DNIC having [Fe^3+^(NO^−^)_2_] or [Fe^+^(NO)_2_] cores capable of antiferromagnetic interactions are characterized by the presence of low-lying excited levels responsible for strong spin-lattice interactions in accordance with the Orbach mechanism [76–79]. At temperatures above 200 K, the EPR signals from these systems can be strongly broadened so that their linewidths, if recorded at ambient temperature, significantly exceed those of DNIC. Therefore, the systems with exchange magnetic interaction are investigated by the EPR method at low temperatures, which vary from 150 K to liquid helium temperature [73, 79].

 The totality of experimental data suggests that it is the model of DNIC with thiol-containing ligands and a Fe^+^(NO^+^)_2_ core {Fe(NO)_2_}^7^ (according to Enemark-Feltham's classification) characterized by low-spin (*S* = 1/2) d^7^configuration and localization of the unpaired electron on the d_*z*2_ orbital that is fully consistent with the EPR data for M-DNIC in the solution. In addition, it provides an explicit explanation for the ability of DNIC with thiol-containing ligands to S-nitrosate thiols both in the presence and in the absence of oxygen.

 At the same time, we do not rule out the possibility that future studies will provide information that might require additional modification or sophistication of this model. It is not excluded that other DNIC models, for example, DNIC with a [Fe^−^(NO^+^)_2_] core ({Fe(NO)_2_}^9^) [80] will prove to be more essential for adequate interpretation of our experimental data. In our opinion, the controversy between our model and the alternative viewpoint according to which electronic and spatial structures of M-DNIC with thiol-containing ligands in the solution must be described in the paradigm of the {Fe(NO)_2_}^9^ structure [66–69] is not yet over and concerns the following fundamental issue. In our opinion, the spatial and electronic structures of crystal DNIC change after their dissolution, namely, the tetrahedron is converted into a plane-square structure as a result of which the {Fe(NO)_2_}^9^ structure (according to the Enemark-Feltham classification characteristic of crystal DNIC) is substituted for {Fe(NO)_2_}^7^, which is more typical of soluble DNIC. Our opponents state that dissolution of DNIC crystals does not influence their electronic and spatial structures. The end to our debates can be put by the results of the crucial test, for example, successful registration of the EPR signal for some monocrystalline DNIC with thiol-containing ligands. By measuring the dependence of the position of this EPR signal in the magnetic field from the angle of the axes of the monocrystal and the direction of the magnetic field in the EPR radiospectrometer, one can obtain the value of the *g*-factor tensor for this signal. If these values correlate with the corresponding values for the EPR *g*-signal of soluble DNIC in frozen solution, our opponents are right; if not, our concepts of soluble DNIC are more verisimilar.

 In conclusion, we think it expedient to state that analysis of electronic and spatial structures of soluble DNIC with thiol-containing ligands not only presents a purely academic interest, but also is a valuable tool for designing directed synthesis of a vast array of medicinal drugs based on water-soluble DNIC with ligands used as NO and NO^+^ donors. It was found, in particular [80–83], that such DNIC do not exert cytotoxic effects by themselves. DNIC with glutathione were used as a basis for the hypotensive drug Oxacom whose single i/v injection to human patients induced long-lasting hypotension [27]. The development, on the basis of DNIC with thiol-containing ligands, of drugs endowed with wound-healing and penile-erective action in animals, is currently under way [24–26]. Our experiments of rats with experimental endometriosis revealed that DNIC strongly eliminate this pathology (in press) and represent an effective remedy against endometriosis in human patients. Of considerable interest are findings illustrating increased elasticity of human erythrocytes after administration of DNIC [21].

 DNIC with thiol-containing ligands are devoid of proapoptotic activity [81, 83]. This concerns, primarily, decomposing DNIC, for example, after treatment of DNIC with iron-chelating agents [81]. Quite probably, in this case the proapoptotic activity of DNIC stimulated is a result of massive release of NO whose interaction with superoxide stimulates enhanced production of peroxynitrite, an extremely cytotoxic compound endowed with apoptotic activity [84]. Apoptosis can also be provoked due to ability of decomposing DNIC to induce S-nitrosation of a vast majority of proteins [85] in the presence of NO^+^-ligands both in the norm and in hypoxia. The data available thus far suggest that the use of DNIC under conditions of their decomposition in cells and tissues is a promising approach, which opens up fresh opportunities for treatment of bacteria- and mycobacteria-borne infectious diseases and may prove to be effective in suppressing malignant growth.

 The results of the most recent research carried out by a group of Italian investigators [86] provided additional support in favour of this hypothesis. It was found that after 4-h treatment of cultured human lung carcinoma MCF7 cells with 0.5 mM S-nitrosoglutathione (GS-NO) the former lost their resistance to doxorubicin. This phenomenon was accompanied by glutathiolation (binding of glutathione to thiol-containing groups of histone proteins) and DNIC synthesis. The authors did not go into detail about the mechanism of histone glutathiolation induced by interaction of tumor cells with GS-NO. At the same time, many authors share an opinion that S-thiolation of an immense variety of proteins is coupled with their S-nitrosation with subsequent substitution of the NO molecule in protein RS-NO for low-molecular thiol and evolution of NO^−^[87–90]. It is not excluded that S-nitrosation of proteins in these cells is not mediated by a reaction of thiol-containing groups of proteins with GS-NO (S-nitrosation), but, rather, by a reaction of thiols with DNIC formed in the cells as nitrosonium ion donors (NO^+^ donation by DNIC to RS-groups).

## Figures and Tables

**Figure 1:**
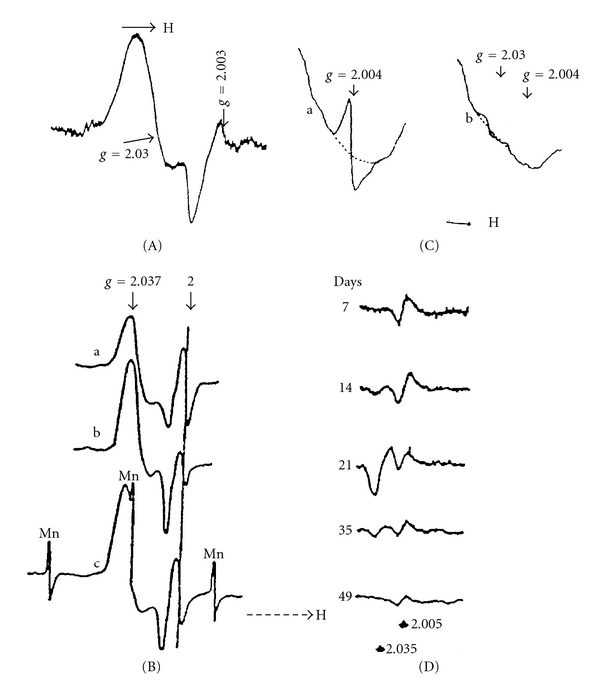
The first recordings of 2.03 signal: dry yeast cells (A) [10]; wet yeast cells and rabbit liver (B, spectra a, and b, c). Three components of hyperfine structure of EPR signal of Mn ions in MgO sample are shown in spectrum c [14]; rat liver carcinoma induced by p-dimethylaminoazobenzene (butter yellow) (C, spectrum b; (a) spectrum from normal liver) [11]; (D) livers from rats maintaining 7, 14, 21, 35, and 49 days on a diet containing butter yellow [13]. Recordings were made at ambient temperature (A, D) or at 77K (B, C).

**Scheme 1:**
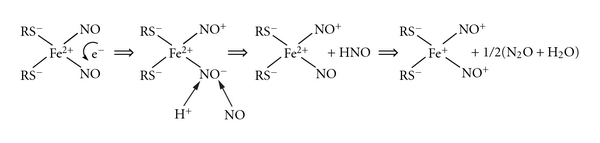


**Figure 2:**
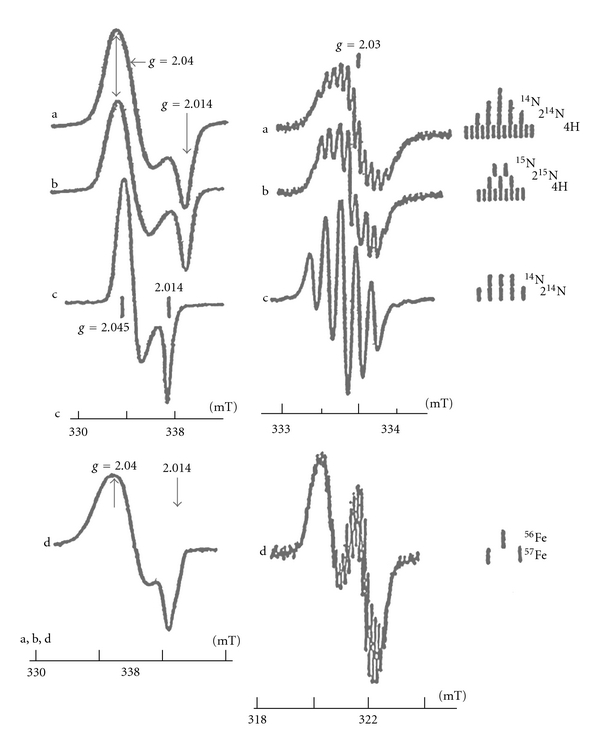
Spectra EPR of 2.03 complex from the solutions of DNIC with cysteine, containing ^14^NO (a) or ^15^NO (b), DNIC with mercaptotriazole (c), or DNIC with cysteine containing ^57^Fe (d). Recordings were made at 77 K (left side) or at ambient temperature (right side). Right side: magnetic field scales are shown separately for EPR signal of DNIC with mercaptotriazole (c) and for EPR signals of DNIC with cysteine containing ^14^NO, ^15^NO, or ^57^Fe (a, b, d).

**Scheme 2:**